# Distinctions in Fine-Scale Spatial Genetic Structure Between Growth Stages of *Picea jezoensis* Carr.

**DOI:** 10.3389/fgene.2018.00490

**Published:** 2018-10-24

**Authors:** Keiko Kitamura, Atsushi Nakanishi, Chunlan Lian, Susumu Goto

**Affiliations:** ^1^Hokkaido Research Center, Forestry and Forest Products Research Institute, Forest Research and Management Organization, Sapporo, Japan; ^2^Asian Natural Environmental Science Center, The University of Tokyo, Tokyo, Japan; ^3^Education and Research Center, The University of Tokyo Forests, Graduate School of Agricultural and Life Sciences, The University of Tokyo, Tokyo, Japan

**Keywords:** coarse woody debris, demographic genetics, kinship, microsatellite, safe-site, seed dispersal, sub-boreal forest

## Abstract

Conifers in northern forests, such as fir and spruce, preferably regenerate on coarse woody debris, including fallen logs, stumps, and snags. In northern Japan, the sub-boreal conifer species *Picea jezoensis* is completely dependent on coarse woody debris for seedling establishment. To understand the fine-scale spatial genetic structure (FSGS) of this species, a 5-ha plot was established in central Hokkaido, and 531 individual trees were categorized into four life-stages (seedling, sapling, juvenile, and mature) on the basis of age and size. The FSGS of the established seedlings and later growth stages was investigated using 11 nuclear simple sequence repeat loci. A STRUCTURE analysis of seedlings and saplings established on fallen logs revealed that genetically related individuals were spatially localized between adjacent logs. We also found a significant FSGS in early life-stages based on a decline in the kinship coefficient calculated between individuals over shorter to longer spatial distances. Furthermore, the estimation of dispersal kernels indicated the frequent occurrence of short-distance seed dispersal. These results indicated that genetically related seedlings and saplings regenerated on the same or nearby fallen logs. In contrast to the results for the early stages, mature-stage trees showed no significant FSGS. We ran a simulation to examine the hypothesis that the FSGS could be eliminated by demographic thinning during life history processes. We calculated values for simulated offspring generated under three sets of conditions; i.e., by removing (i) inbred individuals, (ii) randomly chosen individuals, and (iii) all individuals on the specific fallen logs. However, the results for the FSGS were significant for all simulated data sets. This indicated that inbreeding depression, stochastic loss, or eradication of establishment sites by local disturbances alone could not explain the lack of FSGS among mature-stage trees. Therefore, it is possible that the colonization history of mature trees present on the study site might differ from that of the current offspring.

## Introduction

Sub-boreal conifer forests are widely distributed throughout the northern hemisphere, including North America, northern Europe, and western Siberia, and are mainly dominated by the genera *Picea, Abies*, and *Tsuga*. These coniferous species successfully regenerate at safe sites, which are most often decaying logs and areas of coarse woody debris (CWD) (McCullough, 1948; Harmon, 1987; Suzuki et al., 1987; Nakamura, 1992), and some are so strongly dependent on CWD for their establishment that they are highly susceptible to changes in the environment. Regeneration on CWD is generally a characteristic of cool-temperate to boreal forest conifers in forest ecosystems throughout Europe (Hofgaard, 1993; Bače et al., 2012), the Pacific coast of North America (Harmon and Franklin, 1989), and East Asia (Kubota et al., 1994; Nakagawa et al., 2003). CWD is more beneficial than the soil surface for seedling establishment as it affords better light conditions (Harmon and Franklin, 1989; Maruyama et al., 2004) and fewer pathogens (Cheng and Igarashi, 1987). Previous studies on the successful establishment of tree species on CWD have revealed a number of preferred environmental factors for effective seed capture, germination, survival, and growth (Takahashi et al., 2000; Narukawa et al., 2003). However, in contrast to the numerous studies undertaken on regeneration on CWD, few studies have investigated the demographic processes from seedlings to mature trees for CWD-dependent species.

Recently, the use of molecular markers has enhanced our understanding of the genetic diversity in natural plant populations. Fine-scale spatial genetic structure (FSGS) within wild populations; i.e., the non-random spatial distribution of genotypes (Vekemans and Hardy, 2004), is a consequence of the combined effects of various evolutionary forces such as isolation, drift, selection, and adaptation. These forces are influenced by biotic and abiotic factors, such as mating systems, seed and pollen dispersal, the density of reproductive individuals, and the microenvironmental situation at the establishment sites (Epperson, 1993; Hamrick et al., 1993; Kalisz et al., 2001; Chung et al., 2003). In addition, human activities, such as the selective logging of forest tree species, may reduce the number of reproductive individuals and affect both population gene diversity and FSGS (Lourmas et al., 2007; Carneiro et al., 2011). Demographic processes from early to later life-stages, such as demographic thinning (Kitamura et al., 1997; Ueno et al., 2002; Lian et al., 2008) and generation overlapping (Ueno et al., 2002), may also affect FSGS. In addition, if individuals from different life-stages originate from different reproductive individuals, seed dispersal patterns, or seedling establishment opportunities, the FSGS should differ between life-stages. Thus, studies on FSGS at different life-stages could substantiate the demographic and regenerative processes between life-stages. However, few studies have investigated FSGS in forest tree species with CWD-dependent regeneration. In their survey of *Abies sachalinensis*, a CWD-dependent regeneration species, Lian et al. (2008) investigated FSGS in different growth stages using microsatellite markers. The authors detected a significant FSGS at the seedling stage, possibly attributable to limited seed dispersal, but a decline in FSGS in the later growth stages. They suggest that sib-competition and the density-dependent effect from juvenile to mature stages might lessen the FSGS in *A. sachalinensis*. In contrast to *A. sachalinensis*, which mainly regenerates on CWD but can also regenerate directly on forest soil (Nakagawa et al., 2001), the establishment of *Picea jezoensis* seedlings depends entirely on CWD. This can be explained by the fact that *P. jezoensis* seedlings are more susceptible to snow blight disease than are *A. sachalinensis* seedlings (Cheng and Igarashi, 1990). Furthermore, *P. jezoensis* seedlings were found to successfully establish themselves at more recent areas of CWD owing to their small size (Iijima and Shibuya, 2010). Consequently, a limited number of establishment sites results in the spatially uneven distribution of individuals (Nakagawa et al., 2003). Okada et al. (2015) identified *P. jezoensis* seedlings and saplings established on fallen logs at the same study site as that used by Lian et al. (2008), and they genotyped them using microsatellite markers and monitored survival and death over a 7-year period. They found that individual heterozygosity positively affected seedling longevity in *P. jezoensis*. As a matter of course, the survival or death of genetically related individuals may affect the spatial genetic structure of the later life-stages (Kitamura et al., 2008). However, the previous study by Okada et al. (2015) did not investigate *P. jezoensis* FSGS or seed dispersal patterns, which may be expected to strongly reflect a lack of consistent opportunities for regeneration on CWD. Limited seed dispersal and restriction of establishment sites may reduce the effective number of seed parents, which may induce a strong FSGS; however, the genetic or stochastic death of individuals may weaken the FSGS.

Generally, numerous years of regeneration on spatiotemporally varying areas of CWD leads to the accumulation of a substantial number of different mother trees all contributing to the next generations. The effective number of seed parents affects the FSGS of the offspring, as increases in gene dispersal and density of reproductive individuals reduce the intensity of genetic structure by overlapping “gene shadows” (Oddou-Muratorio and Klein, 2008). In fact, the intensity of the FSGS as expressed statistically as *Sp* is the reciprocal of the neighborhood size under certain conditions (Vekemans and Hardy, 2004). Therefore, the more the number of mother trees contributes to reproduction, the weaker the FSGS becomes. In this regard, the population of mature *P. jezoensis* individuals consists of overlapping generations established over many years, with the long life-span of this species (Hiura et al., 1996) thereby resulting in a weak FSGS. Thus, the study of FSGS verifies the conservation of genetic diversity as well as the reproductive process of the local population.

In the present study, we estimated the FSGS in *P. jezoensis* at four different growth stages, seedling, sapling, juvenile, and mature trees. Our working hypotheses are as follows; (1) strong restriction of safe-sites decreases the number of mother trees contributing to offspring and results in a strong FSGS at the early demographic stages and (2) accordingly, the FSGS at the early demographic stage should be weaker at the later demographic stages. Finally, we examined the factors affecting the FSGS, such as inbreeding depression, stochastic loss of individuals, and the eradication of establishment sites by local disturbances using simulation studies.

## Materials and Methods

### Study Site and Genotyping

The 5-ha study site (200 m × 250 m) was established at the fluvial terrace of the Iwanazawa Forest Reserve, University of Tokyo Hokkaido Forest (UTHF) in central Hokkaido (43°13′N, 142°34′E), Japan. The FSGS of co-occurring tree species; namely, *Fraxinus mandshurica* var. *japonica* (Goto et al., 2006), *Cercidiphyllum japonicum* (Sato et al., 2006), and *Betula maximowicziana* (Uchiyama et al., 2006) were investigated in a series of studies in the Iwanazawa Forest Reserve. Moreover, Lian et al. (2008) revealed the spatial genetic structure of CWD-dependent *A. sachalinensis*. The present study deals with the other species with CWD-dependent regeneration, *P. jezoensis*, on the same study plot.

In this study site, there were 112 fallen logs the diameters of which were larger than 20 cm at the base. Fallen logs were regarded as a major component of CWD in this forest. We measured individual *P. jezoensis* plants and identified four growth stages in the study plot: mature, juvenile, sapling, and seedling. We observed all 123 mature *P. jezoensis* individuals with a diameter at breast height (dbh) ≥20 cm, and all 39 juveniles with a dbh ≥5 cm but <20 cm. Individual trees with a dbh <5 cm were differentiated into two categories by age as determined by the number of bud scale vestiges. For saplings and seedlings, we selected half of the individuals established on fallen logs in this study, so that 190 saplings aged ≥3 years and 179 seedlings aged <3 years were included in this study. The locations of the areas of CWD and individual trees were identified using a digital compass (LaserACE 300, TimberTech), and their needle leaf tissues were collected for DNA analysis.

DNA was extracted from dried needle leaf samples from 531 individuals using a DNeasy Plant Mini kit (Qiagen). The following 11 microsatellite loci, *GC1, GD1, GG3* (Pfeiffer et al., 1997), *PaGB3, PgGB5* (Besnard et al., 2003), *EATC1E03, EATC2G05* (Scotti et al., 2002), *Pj4, Pj8, Pj22*, and *Pj24* (Okada et al., 2015) were analyzed using a Multiplex PCR Kit (Qiagen) together with a 3130xl Genetic Analyzer and GeneMapper ver. 4.0 (Thermo Fisher Scientific Inc.).

### Data Analyses

The genetic diversity of the four growth stages was calculated using FSTAT ver. 2.3.9.2 (Goudet, 1995). *F*_IS_ was also calculated considering the null alleles using INEst (Chybicki and Burczyk, 2009). Deviations from the Hardy-Weinberg equilibrium (HWE) for the mature stage at each locus and the overall loci were tested using the Markov chain method with GENEPOP (Raymond and Rousset, 1995). The null allele frequency of each locus was estimated using CERVUS ver. 2.0 (Marshall et al., 1998). To evaluate the associations between loci in mature trees, we conducted an exact test of linkage disequilibrium using GENEPOP.

To test for a temporal Wahlund effect, which may be due to differences between reproductive individuals among growth stages, pairwise population differentiation values between all pairs of stages were calculated using *F*_ST_ from an AMOVA (Excoffier et al., 1992; Michalakis and Excoffier, 1996), which is equivalent to Weir and Cockerham (1984) using GENODIVE (Meirmans and Van Tienderen, 2004).

We used four approaches to evaluate the FSGS at the four growth stages, and then examined the factors affecting the FSGS dynamics during the life history process.

First, the local distribution of genetic variation was examined by multilocus model-based cluster analysis using STRUCTURE 2.3.4 (hereafter, STRUCTURE) (Pritchard et al., 2000) for all 531 individuals. All of the runs consisted of 200,000 Markov chain Monte Carlo (MCMC) generations, after a burn-in period of 100,000 iterations. Twenty runs were performed for each number of clusters (*K*), ranging from 1 to 10. To determine the most likely value for *K*, we first used the methods suggested in the original description of STRUCTURE, which involves comparing the log probability to the data (ln Pr [*X*|*K*]) for each run to evaluate the genetic structure. Then, we calculated Δ*K* (Evanno et al., 2005) to determine the optimal value of *K*. Subsequently, we chose the most appropriate *K* (Pritchard et al., 2000) for the inferred clustering interpretation.

Second, the FSGS at each growth stage was evaluated using the kinship coefficient, *F*_ij_ (coancestry) (Loiselle et al., 1995). In the case of two individuals, *i* and *j, F*_ij_ can be defined as *F*_ij_ = (*Q*_ij_–*Q*_m_)/(1–*Q*_m_), where *Q*_ij_ is the probability of identity in state between two randomly chosen genes from *i* and *j*, and *Q*_m_ is the average probability of identity by state between two randomly chosen individuals from the reference population. Assuming that the reference population is the parental generation, *F*_ij_ is expected to have values derived from the pedigree information (i.e., 0.125 for half-siblings [sibs] and 0.25 for full-sibs) (Hardy and Vekemans, 2002). For the analyses of seedlings, saplings, and juveniles (hereafter, these three stages are collectively referred to as adolescents), we assumed the 123 mature trees to be the reference population. When an allele unique to the adolescent stage was observed, one of its haploid genes was incorporated into the reference population to allocate the low frequency for the allele into the reference population. The reference population was used to calculate average *F*_ij_ values for all four stages for each of the 20 continuous distance classes at 10 m intervals from 0–10 to 190–200 m. The significance of the average *F*_ij_ values was then assessed using permutation tests (with 1000 permutations), in which spatial distances were permuted randomly among pairs of individuals. Furthermore, to compare the intensity of the FSGS among the four stages, we calculated *Sp*, a parameter indicating FSGS intensity, using the formula *Sp* = *b*_F_/(*F*_1_–1). In this formula, *b*_F_ is the slope of *F*_ij_ against the logarithm of the distance between individuals, and *F*_1_ is the average kinship coefficient among individuals in the first distance class (i.e., 0–10 m) in this study. The value for *b*_F_ was estimated based on the regression of all spatial distances between individuals, and the significance level was calculated using 1000 permutation tests. All the FSGS-related calculations were performed using SPAGEDI ver. 1.4 (Hardy and Vekemans, 2002).

Third, we evaluated the effect of inbreeding on demographic change as follows. We calculated the average values of individual inbreeding coefficients (*F*_is_) as a kinship coefficient between genes within each individual (Hardy and Vekemans, 2002) at each of the four growth stages, and then examined their significance. Coefficients for all four stages were calculated using the same reference population as used for the FSGS analyses. These calculations were performed using SPAGEDI ver. 1.4.

Fourth, NM+ software (Chybicki and Burczyk, 2010) was used to evaluate the pollen and seed dispersal kernels for seedlings and saplings using the neighborhood model approach, based on the multilocus genotypes of mature trees, seedlings, and saplings (Burczyk et al., 2006). We applied the following exponential-power function, characterized by the parameters *a* and *b* (Clark, 1998):

$$P(r)=\left\{\frac{b}{2\pi a^{2}\Gamma \;\left(\frac{2}{b}\right)}\right\}e^{-{\left(\frac{r}{a}\right)}^{b}}$$

where *r* is the pollen or seed dispersal distance, Γ is the gamma function, *a* is a scale parameter, and *b* is the shape parameter affecting the fat tail of the dispersal curve (Austerlitz et al., 2004). Assuming all 123 mature trees to be neighbors and setting selfing rates to zero, we estimated the parameters *a* and *b*, immigration rates, and average dispersal distances for pollen and seeds for seedling and sapling generations. We incorporated mature tree dbh values as a predictor of reproductive success, and null allele frequencies to the estimation of the kernels.

Finally, to examine whether demographic thinning by inbreeding depression, stochastic loss, or both during the transition from adolescent to mature stage affected the FSGS among mature trees, we simulated seedling and sapling deaths under three sets of conditions. We first combined all 179 seedlings and 190 saplings into a dataset of 369 offspring. We then generated the following datasets. (1) To examine the deaths of inbred offspring; i.e., death by inbreeding depression, we generated a set consisting of 123 offspring with low *F*_is_ values, assuming the death of the top 246 offspring based on high *F*_is_ values. (2) To examine the deaths of random offspring, we randomly extracted 123 individuals from the offspring data without replacement and repeated this procedure 10 times to generate 10 different datasets. This randomization procedure was conducted using Visual Basic for Applications. In the first and second simulations, the number (123) of surviving offspring was the same as that of mature trees. (3) To determine the effects of local disturbances such as flooding, which may eradicate all offspring on specific CWD, we generated six datasets by excluding all offspring on CWD with (i) the largest number of offspring; (ii) the largest and second largest number of offspring; (iii) the largest, second, and third largest number of offspring; (iv) the largest, second, third, and fourth largest number of offspring; (v) the largest, second, third, fourth, and fifth largest number of offspring; and (vi) more than eight offspring, resulting in 250, 232, 216, 200, 186, and 122 surviving individual data sets, respectively. The number of datasets from (vi) was approximately the same as that of the mature trees. We calculated test statistics for the FSGS of the simulated offspring datasets (1, 2, and 3), to examine whether the FSGS was significant in each dataset. In these simulation-based analyses, all the statistical calculations for the FSGS of actual mature trees, and actual and simulated offspring were determined using the same methods as previously mentioned for the examination of FSGS at each growth stage, but without the reference population.

## Results

### Genetic Diversity

The genetic diversity (*H*_S_) of each growth stage ranged from 0.743 to 0.751, and the allelic richness (based on 37 individuals) ranged from 14.01 to 15.19 (Table 1). The juvenile stage showed the highest values for both parameters, although the values for these parameters were similar among the four growth stages. The inbreeding coefficient (*F*_IS_) was the highest and lowest in the seedling and mature stages (0.241 and 0.113), respectively. This was also observed for the *F*_IS_ values (0.074 and 0.024 in seedling and mature tree stages, respectively) estimated using the Bayesian approach taking into account the null alleles (i.e., using the INEST software). The 11 loci were polymorphic in mature trees, with 3 to 46 alleles per locus and mean *H*_E_ values of 0.743 (Supplementary Table 1). The *F*_IS_ values for each locus ranged from -0.054 to 0.286, with an average of 0.112 across all loci. Significant deviations from the HWE were observed at five loci: *GD1, Pj22, Pj24, EATC1E03*, and *PaGB3* (*p* < 0.05 after Bonferroni correction), which all had high null allele frequencies, ranging from 0.090 to 0.185. Therefore, these loci were excluded from the calculations of *F*_ij_ and *F*_is_. There was no significant linkage disequilibrium between any pair of loci analyzed.

**Table 1:** Genetic diversity of each growth stage of *Picea jezoensis*.

Stage	*H*_O_	*H*_S_	*F*_IS_	*R*_S_	*F*_IS_null_
Mature	0.659	0.743	0.113	14.96	0.024
Juvenile	0.626	0.751	0.166	15.19	0.062
Sapling	0.643	0.743	0.134	14.01	0.057
Seedling	0.564	0.743	0.241	14.13	0.074


*H_O_, observed heterozygosity; H_S_, gene diversity; F_IS_, inbreeding coefficient; R_S_, allelic richness calculated based on 37 individuals; F_IS_null_, F_IS_ calculated using Bayesian approach considering null alleles.*

Pairwise *F*_ST_ values between growth stages were calculated using 11 and 6 loci without the above-mentioned significant deviations from the HWE loci (Table 2). Genetic differentiation was not detected between mature and juvenile stages. However, the mature stage was significantly differentiated from the sapling and seedling stages. The juvenile stage was also differentiated from saplings and seedling stages, but the *F*_ST_ values obtained by 6 loci were not significantly different between juveniles and saplings. Differentiation between saplings and seedlings was also significant. This result indicated that a genetic differentiation had occurred, with a temporal Wahlund effect detected between growth stages.

**Table 2:** Pairwise differentiation between four growth stages using *F*_ST_ from AMOVA using 11 and 6 loci.

	Mature	Juvenile	Sapling	Seedling
Mature	-	0.000^n.s.^	0.004^∗∗^	0.005^∗∗^
Juvenile	-0.001^n.s.^	-	0.009^∗∗^	0.009^∗∗^
Sapling	0.005^∗∗^	0.005^n.s.^	-	0.003^∗^
Seedling	0.007^∗∗^	0.007^∗^	0.005^∗^	-


*Upper and lower triangles, results of 11 and 6 excluded loci, respectively, with high frequency of null allele. ^*n.s*.^Not significant; ^∗^*p* < 0.05 and ^∗∗^*p* < 0.01 after Bonferroni correction.*

### Fine-Scale Spatial Genetic Structure

**FIGURE 1:**
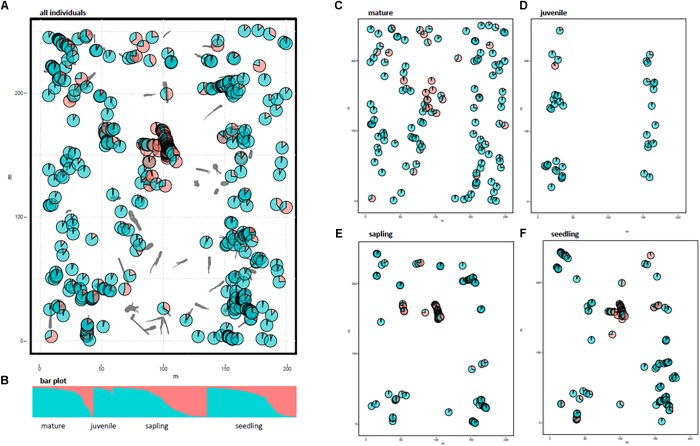
Distribution of two cluster components of 531 individuals using STRUCTURE analysis. Gray bars indicate coarse woody debris (CWD). Pie charts represent the cluster components of each individual plant. Different colors indicate different clusters (*K* = 2); Cluster 1 in red and Cluster 2 in green. **(A)** All 531 individuals, **(B)** bar plot for *K* = 2, **(C–F)**, cluster components for each growth stage without CWD locations.

The spatial genetic structure was revealed using STRUCTURE analysis (Figure 1). We chose *K* = 2 as the most appropriate number of clusters to distinguish any local assemblage based on the rate of change in the log probability of the data (ln Pr [*X*|*K*]) between successive *K* values (Supplementary Figure 1). The distribution of each cluster component for each individual was mapped using the location of each area of CWD (Figure 1). Allele frequency divergence between 2 clusters was 0.028. The mean value of *F*_ST_ for Cluster 1 (red in Figure 1) and Cluster 2 (green in Figure 1) was 0.076 and 0.0086, respectively. The localized distribution of Cluster 1 was observed at the center of the study plot (red in Figure 1A). The spatial distribution of individuals differed between growth stages (Figures 1C–F). Mature trees were evenly distributed in the study plot, but juveniles were extremely localized; the spatial distribution of any cluster was insignificant for the mature and juvenile stages (Figures 1E,F). Saplings and seedlings were restricted to the areas of CWD and showed a spatially distinct concentration for Cluster 1 (Figures 1E,F). We calculated the average *F*_ij_ values of seedlings and saplings within Cluster 1 to examine the relationships comparable to half or full-sibs. We chose seedlings and saplings with probabilities of belonging to Cluster 1 higher than 0.9, which resulted in a final population of 59 seedlings and 59 saplings. We then calculated the *F*_ij_ values between 1711 pairs for seedlings and saplings, respectively. The average *F*_ij_ for seedlings and saplings for Cluster 1 was 0.025 and 0.011, respectively.

**FIGURE 2:**
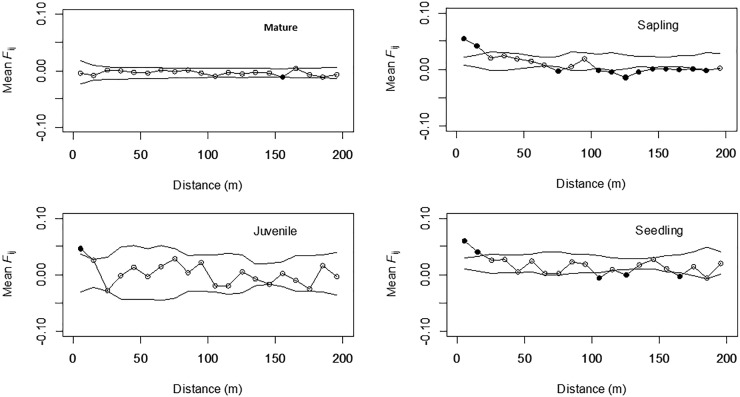
Mean *F*_ij_ (kinship coefficient) for each growth stage in *Picea jezoensis*. Dotted lines, 95% confidence interval envelopes; ^∗^significance level, *p* < 0.05.

Correlograms of the average *F*_ij_ values for the four different growth stages are shown in Figure 2. Significantly positive values were observed in the shorter distance classes for seedlings (0–10 and 10–20 m), saplings (0–10 and 10–20 m), and juveniles (0–10 m), but the values were either not significant or significantly negative in the longer distance classes for these three stages; however, this tendency was not detected for mature trees. The *b*_F_ values for juveniles (-0.008), saplings (-0.013), and seedlings (-0.011) were all significant (permutation test, *p* < 0.05), but were not significant for mature trees (-0.001, Table 2). The *Sp* values for mature trees (0.001) were much lower than those for juveniles (0.009), saplings (0.014), and seedlings (0.012). This indicated that the FSGS was stronger in the adolescents than in the mature trees.

### Inbreeding Detected at Each Growth Stage

The average value of the individual inbreeding coefficient (*F*_is_) at all four growth stages was significantly high (permutation test after Bonferroni correction, Table 3). However, the average value for seedlings (0.228, *p* < 0.005) was the highest, and the values then decreased with life history for saplings (0.094, *p* < 0.005), juveniles (0.072, *p* < 0.05), and mature trees (0.034, *p* < 0.05).

**Table 3:** Genetic parameters of inbreeding and spatial genetic structure for the different growth stages of *Picea jezoensis.*

Stage	*F*_is_	*F*_1_	*b*_F_	(SE)	*Sp*
Mature	0.034^∗^	-0.004	-0.001	(0.001)	0.001
Juvenile	0.072^∗^	0.046	-0.008	(0.006)^∗^	0.009
Sapling	0.094^∗^	0.055	-0.013	(0.002)^∗^	0.014
Seedling	0.228^∗^	0.061	-0.011	(0.004)^∗^	0.012


*F_is_, individual inbreeding coefficients; F_1_, average F_ij_ for the distance class of 0–10 m; b_F_, regression slope calculated by regression of F_ij_ against the logarithm of the distance between individuals; Sp, a parameter indicating the intensity of the fine-scale spatial genetic structure (Vekemans and Hardy, 2004). The standard error (SE) was estimated using the jackknife procedure. The significance of F_is_ and b_F_ was assessed by permutation tests. ^∗^p < 0.05.*

### Estimated Patterns of Seed and Pollen Dispersal

Based on the neighborhood model and using exponential power functions as dispersal kernels for both pollen and seeds, the scale parameter (*a*), shape parameter (*b*), average dispersal distance (*d*), and immigration rate (*m*) were calculated to be 81.816, 3.097, and 59.9 m and 86.6%, respectively, for pollen dispersal in the seedling stage. Furthermore, the corresponding values for seed dispersal were 3.823, 0.571, and 40.6 m and 74.4%, respectively, for the seedling stage (Table 4). The above parameters for pollen dispersal in the sapling stage were 0.010, 0.251, and 71.6 m and 82.4%, respectively, while those for seed dispersal were 2.710, 0.564, and 30.5 m and 61.2%, respectively (Table 4).

**Table 4:** Parameters of pollen and seed dispersal kernels at the seedling and sapling stages, estimated according to the neighborhood model approach using exponential power functions.

	Seedling				Sapling			
		
Parameter	Pollen	(SE)	Seed	(SE)	Pollen	(SE)	Seed	(SE)
*a*	81.816		3.823		0.010		2.710	
*b*	3.097	(4.130)	0.571	(0.198)	0.251	(0.212)	0.564	(0.145)
*s*	0.000				0.000			
*d*	59.860	(11.9)	40.558	(10.5)	71.633	(80.6)	30.522	(5.1)
*m*	0.866	(0.051)	0.744	(0.033)	0.824	(0.045)	0.612	(0.036)
*Sigma (dbh)*	1.540	(0.807)	1.211	(0.268)	1.399	(0.459)	1.217	(0.183)


*a, scale parameter; b, shape parameter; s, selfing rate; d, average distance (m); m, immigration rate; sigma (dbh), selection gradient for dbh (diameter at breast height) of mature trees as a predictor of reproductive success; SE, standard error. Selfing rates were set to zero.*

### FSGS of Simulated Offspring

Fine-scale spatial genetic structure values were significant for seedlings and saplings observed on the study site, and for all the sets of simulated offspring under the three conditions, except for mature trees (Supplementary Table 2). For simulated offspring selected under the condition of death by inbreeding depression, although the average *F*_is_ value was negative and significantly low (permutation test, *p* < 0.001), the *F*_1_ and *b*_F_ values remained significantly high (permutation test, *p* < 0.001). For the sets of offspring simulated under the two sets of conditions related to random death and eradication of all the offspring on specific CWD, the average *F*_is_, *F*_1_, and *b*_F_ values for all the data sets were significantly high (permutation test, *p* < 0.001). Furthermore, the *Sp* values (0.004–0.010) for all the sets of simulated offspring were higher than those of mature trees (0.001).

## Discussion

### Significant FSGS in Adolescent Stages

The STRUCTURE analysis indicated that adjacent areas of CWD accommodated genetically related progeny at the center of the study plot (Figures 1E,F). This might reflect limited seed dispersal capacity and a limited number of established sites (CWD), both of which decrease the effective number of seed parents and, consequently, strengthen the FSGS in this species. Moreover, correlograms of the average *F*_ij_ value for adolescents showed a significant FSGS for short distance classes (i.e., within a specific area of CWD and between adjacent areas of CWD, Figure 2). It was comparable to the significance of the regression slope (*b*_F_), indicating that the genetic clustering of adolescents was stronger than that of mature trees (Table 3). Although *P. jezoensis* is wind-pollinated and anemochorous, it demonstrates FSGS in its early life-stages, which could be attributed to the limited gene dispersal and small number of establishment sites. The *F*_1_ (mean *F*_ij_ at 0–10 m distance class) values (0.046–0.061) for adolescents were much lower than those expected for half-sibs (0.125). Moreover, the average *F*_ij_ values for seedlings and saplings for Cluster 1 in the STRUCTURE analysis (0.025 and 0.011, respectively) were also much smaller than the kinship coefficients expected for half-sibs. The significant FSGS and low *F*_1_ values for adolescents indicated that spatially limited seed dispersal should generate a half-sib family structure around seed parents, but this structure may be weakened by the overlapping of seed shadows.

Even in the case of anemochory, the seed shadow of each mother tree would create a spatial genetic structure during the initial regeneration process (Uchiyama et al., 2006). The proportion of seed and pollen flow from outside the plot was high in both the seedling and sapling stages, indicating long-distance seed and pollen dispersal. However, seed dispersal was limited locally because the average distances of seed dispersal at the seedling (40.6 m) and sapling (30.5 m) stages were shorter than the average distances for pollen dispersal. Similar results were obtained from a study of the anemochorous species, *A. sachalinensis*, at the same study site, for which the average distance of seed dispersal was 23.7 m (Lian et al., 2008). It is probable that frequent seed dispersal over short distances may have induced the significant FSGS observed for the adolescent stages. As the proportion of seed flow from outside the plot and the mean distances of seed dispersal for *P. jezoensis* detected in this study were higher and longer than those (18.7% and 23.7 m, respectively) for *A. sachalinensis* in the same plot (Lian et al., 2008), the distance of seed dispersal is expected to be longer in *P. jezoensis* than in *A. sachalinensis*. However, the *Sp* values at the seedling, sapling, and juvenile stages for *P. jezoensis* were higher than those for *A. sachalinensis* (0.004, 0.004, and 0.004, respectively). The stronger FSGS for *P. jezoensis* may be due to its stronger dependency on CWD for establishment than that of *A. sachalinensis*.

### Evidence of Inbreeding Among Early Life Stages

The genetic diversity parameters were similar among the four growth stages (Table 1). In contrast, the average *F*_is_ value was high at the seedling stage and decreased for the later growth stages (Table 3). Husband and Schemske (1996) suggested that outcrossing species express much of their inbreeding depression either early, at the time of seed development, or later during the growth and reproductive stages. The decrease in average *F*_is_ value with life history process might be influenced by inbreeding depression at the growing stages after seedling establishment. Our previous study, based on the same population examined in the present study, indicated that multilocus heterozygosity had an effect on seedling longevity (Okada et al., 2015). Some cross-experiment studies of *Picea* species have shown that growth in height decreased as the kinship coefficient increased (Doerksen et al., 2014). Therefore, it is likely that not only decreasing growth in height but also increasing mortality due to inbreeding depression may affect *F*_is_ values during the life history process of *P. jezoensis*. Additionally, inbreeding depression might be one of the reasons for the weakening of the FSGS during the progression from juveniles to mature trees.

### Inbreeding Depression, Stochastic Loss, or Local Disturbances Alone Did Not Explain the Decline in FSGS in Mature Trees

In the present study, we found a significant FSGS in seedlings, saplings, and juveniles, but not in mature trees. Thus, our two working hypotheses were confirmed by this study. First, the decline in FSGS over the life history was previously reported for various tree species (Hamrick et al., 1993; Epperson and Alvarez-Buylla, 1997; Lian et al., 2008; Schroeder et al., 2014; Ganzhorn et al., 2015), and has been attributed to the demographic thinning of individuals. Second, the previously mentioned inbreeding depression might also have weakened the FSGS in our study. Furthermore, the *Sp* value of mature *P. jezoensis* trees (0.001) in this study was much lower than that of other tree species that relay on wind-pollination and wind-dispersal of seeds (0.002–0.011) (Vekemans and Hardy, 2004), and that of the CWD-dependent *A. sachalinensis* (0.003) (Lian et al., 2008).

However, according to the results of the simulations under the conditions of death by inbreeding depression, stochastic loss, and local disturbance, the FSGS values for all simulated offspring datasets were significant (Supplementary Table 2). These results appear to indicate that demographic thinning by the stochastic loss of individuals, removal of specific areas of CWD by local disturbances, or via the inferior fitness of inbred individuals, did not reduce the FSGS in the adolescent life stages of *P. jezoensis*. In conclusion, we cannot attribute the decline in the FSGS throughout the life history of *P. jezoensis* to demographic thinning alone. It is probable that the regeneration processes responsible for each generation have changed significantly over long periods. Thus, not only demographic thinning but also other factors, such as differences in colonization histories between the mature and offspring populations, and seeds derived from multiple sources, should be considered in explaining the low *Sp* values and lack of FSGS in mature *P. jezoensis* populations.

We do not currently have any concrete evidence to explain the observed lack of FSGS in the mature stage. However, it is most probable that the mature population consists of individuals derived from multiple origins by long-distance propagule dispersal. This is likely if the mature individuals are a founder population established after a strong disturbance, and it must be remembered that the study site is located on a floodplain along a creek. We speculate that immediately after the vegetation was cleared by natural disturbances such as flooding, no effective mother trees remained in the adjacent area, but substantial CWD was available for *P. jezoensis* establishment; i.e., it was an ecologically empty site.

### Insights Into Regeneration Processes Using FSGS Analysis

Figures 1C–F clearly shows that the spatial distribution of individuals differed among growth stages. For example, mature trees spread over the whole study site (Figure 1C), whereas there were relatively small numbers of juveniles restricted to either end of the study site (Figure 1D). Saplings and seedlings showed similar localization patterns at each area of CWD (Figures 1E,F). The results of FSGS analysis differed among size classes, namely, a significant FSGS was observed in the proximate distance classes of seedlings and sapling stages (Figure 2). This might reflect limited seed dispersal and establishment sites in the present forest. The distribution of each growth stage reflects the existence of ecological empty patches at that time of regeneration. As the study site is located on the fluvial plain of Iwanazawa creek, the widely scattered distribution of mature trees (Figure 1C) suggests that these individuals were established in an ecological empty patch created by a strong natural disturbance such as the clearance of the forest floor by flooding. The longevity of *P. jezoensis* (100–160 years old) also suggests that the substantial number of areas of CWD accumulated after the initial establishment and regeneration of the younger mature trees. In addition, an artificial disturbance appears to have occurred at the study site in the past. The distribution of juveniles reflected the track of an abandoned forest railway and the present-day forest road. As there are large broadleaf trees growing at the center of the study plot, it seems that few areas of CWD are available for the regeneration of the present juveniles. The limited area available for regeneration may lead to a weak but significant FSGS in the juvenile stage in spite of its small sample size (Figure 2). Recently created areas of CWD have been revealed as suitable substrates for the regeneration of *P. jezoensis* (Iijima and Shibuya, 2010). In fact, similar but, in some part, slight differences were observed in the genetic distribution between the saplings and seedlings. This reflect differences in the time of CWD production.

In conclusion, the number and distribution of establishment sites, such as CWD, may be attributable to the FSGS in each generation of *P. jezoensis*. Moreover, our FSGS simulations suggested that present mature individuals may have been established by different disturbance regimes compared to the other demographic stages. It might be one of the characteristics of CWD-dependent tree species that different genetic variations could be created through spatiotemporal differences in the production of areas of CWD.

## Author Contributions

KK, CL, and SG designed the study and interpreted the results. KK was responsible for the laboratory work. KK and AN analyzed the data. All authors contributed to the final version of the manuscript.

## Conflict of Interest Statement

The authors declare that the research was conducted in the absence of any commercial or financial relationships that could be construed as a potential conflict of interest.

## Abbreviations

CWD: coarse woody debris
FSGS: fine-scale genetic structure
