# Supplementary material for: Distinctions in Fine-Scale Spatial Genetic Structure Between Growth Stages of Picea jezoensis Carr
Source: Front Genet. 2018 Oct 24;9:490. doi: 10.3389/fgene.2018.00490 (PMC6207582; doi:10.3389/fgene.2018.00490)
Supplement: Supplementary file 1 [file Data_Sheet_1.PDF]

Supplementary Figure 1

Distinction in the Fine-scale Spatial Genetic Structure between Growth Stages of *Picea jezoensis* Carr.

Keiko Kitamura, Atsushi Nakanishi, Chunlan Lian, Susumu Goto*

*** Correspondence:** Susumu Goto: gotos@ uf.a.u-tokyo.ac.jp

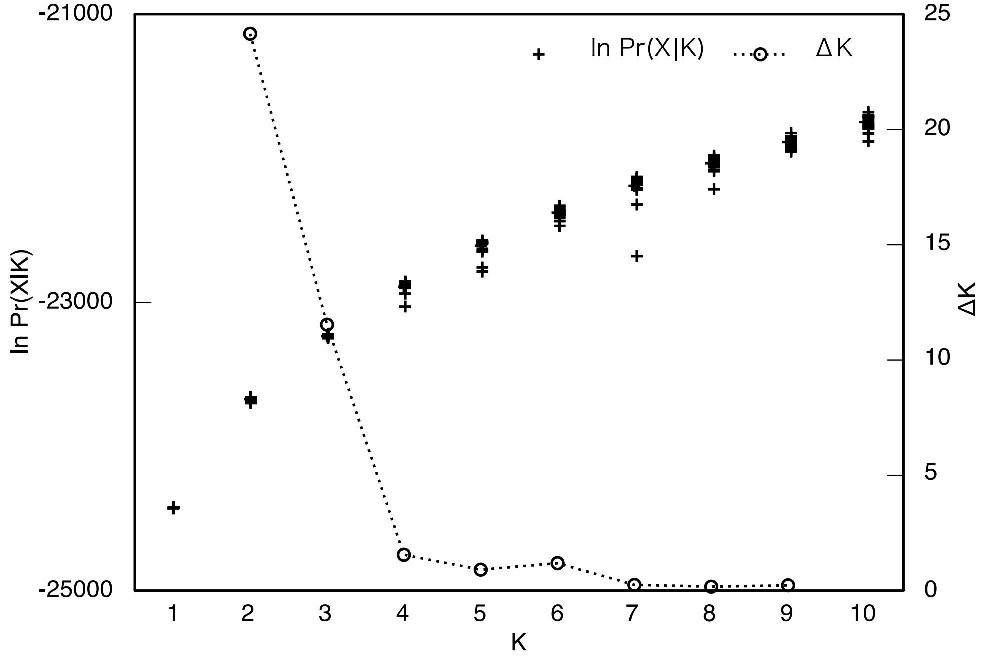

Supplementary Figure 1. Log probability (ln Pr [X|K]) and Δ*K* for STRUCTURE analyses.
