# Supplementary material for: Distinctions in Fine-Scale Spatial Genetic Structure Between Growth Stages of Picea jezoensis Carr
Source: Front Genet. 2018 Oct 24;9:490. doi: 10.3389/fgene.2018.00490 (PMC6207582; doi:10.3389/fgene.2018.00490)
Supplement: Supplementary file 2 [file Data_Sheet_2.PDF]

Supplementary Table 1

Distinction in the Fine-scale Spatial Genetic Structure between Growth Stages of *Picea jezoensis* Carr.

Keiko Kitamura, Atsushi Nakanishi, Chunlan Lian, Susumu Goto*

*** Correspondence:** Susumu Goto: gotos@ uf.a.u-tokyo.ac.jp

Supplementary Table 1. Genetic diversity at the 11 polymorphic microsatellite loci for mature stage in the 5-ha plot.

Locus	k	H_O	H_E	F_{IS}	
Pj8	46	0.935	0.968	0.034	
Pj4	40	0.837	0.952	0.121	
GD1	24	0.772	0.929	0.169	*
GG3	9	0.683	0.648	-0.054	
Pj22	16	0.862	0.923	0.066	*
Pj24	39	0.699	0.947	0.262	*
EATC1E03	6	0.488	0.609	0.199	*
EATC2G05	3	0.260	0.288	0.097	
GC1	21	0.893	0.918	0.027	
PaGB3	10	0.431	0.604	0.286	*
PgGB5	8	0.390	0.382	-0.021	
Mean	20.2	0.659	0.743	0.112	*
S.E.	4.4	0.066	0.071		

k, the number of alleles; H_O, observed heterozygosity; H_E, expected heterozygosity; F_{IS}, inbreeding coefficient $^{*}P < 0.05$.
