# Supplementary material for: Distinctions in Fine-Scale Spatial Genetic Structure Between Growth Stages of Picea jezoensis Carr
Source: Front Genet. 2018 Oct 24;9:490. doi: 10.3389/fgene.2018.00490 (PMC6207582; doi:10.3389/fgene.2018.00490)
Supplement: Supplementary file 3 [file Data_Sheet_3.PDF]

Supplementary Table 2

Distinction in the Fine-scale Spatial Genetic Structure between Growth Stages of *Picea jezoensis* Carr.

Keiko Kitamura, Atsushi Nakanishi, Chunlan Lian, Susumu Goto*

*** Correspondence:** Susumu Goto: gotos@ uf.a.u-tokyo.ac.jp

Supplementary Table 2. Individual inbreeding coefficients and parameters for the fine-scale spatial genetic structure of actual mature trees and offspring, and simulated offspring generated by mortality under three hypothetical simulations.

	N	F_{is}	F_1	b_F	Sp
Actual mature trees	123	0.038 *	-0.001 ^{ns}	-0.0012 ^{ns}	0.001
Actual offspring	369	0.142 ***	0.028 ***	-0.0079 ***	0.008
Simulated offspring					
Hypothesis					
Deaths of the 246 offspring with the highest $F$is values	123	-0.130 ***	0.013 ***	-0.0044 ***	0.004
Deaths of randomly extracted 246 offspring					
Simulation 1	123	0.149 ***	0.023 ***	-0.0064 ***	0.007
Simulation 2	123	0.181 ***	0.030 ***	-0.0098 ***	0.010
Simulation 3	123	0.159 ***	0.023 ***	-0.0066 ***	0.007
Simulation 4	123	0.131 ***	0.020 ***	-0.0057 ***	0.006
Simulation 5	123	0.151 ***	0.034 ***	-0.0088 ***	0.009
Simulation 6	123	0.152 ***	0.024 ***	-0.0080 ***	0.008
Simulation 7	123	0.115 ***	0.023 ***	-0.0057 ***	0.006
Simulation 8	123	0.123 ***	0.030 ***	-0.0093 ***	0.010
Simulation 9	123	0.150 ***	0.029 ***	-0.0091 ***	0.009
Simulation 10	123	0.154 ***	0.025 ***	-0.0075 ***	0.008
Deaths of all the offspring on the designated CWDs with					
the largest number of offspring	250	0.173 ***	0.024 ***	-0.0063 ***	0.006
the largest and the second largest numbers	232	0.170 ***	0.022 ***	-0.0060 ***	0.006
the largest, and the second and the third largest numbers	216	0.170 ***	0.020 ***	-0.0056 ***	0.006
the largest, and the second, third and fourth largest numbers	200	0.155 ***	0.018 ***	-0.0051 ***	0.005
the largest, and the second, third, fourth and fifth largest numbers	186	0.155 ***	0.018 ***	-0.0052 ***	0.005
more than eight offspring	122	0.178 ***	0.013 **	-0.0046 ***	0.005

N, the number of individuals; F_{is}, individual inbreeding coefficients (computed as kinship coefficients between genes within individuals); F_1, average F_{ij} at the distance class from 0 to 10 m; b_F, regression slope calculated by regression of F_{ij} against logarithm of the distance between individuals; and Sp, a parameter indicating intensity of fine-scale spatial genetic structure. The significance of b_F was assessed by permutation tests $^{*}P < 0.05$, $^{**}P < 0.01$, $^{***}P < 0.001$.
